# No particularly negative impact of the Covid-19 pandemic on the mental health of individuals with higher levels of childhood trauma

**DOI:** 10.3389/fpsyt.2025.1452732

**Published:** 2025-04-04

**Authors:** Elisabeth M. Weiss, Andreas Fink, Ilona Papousek, Silvia Exenberger-Vanham, Astrid Lampe, Verena Dresen, Markus Canazei

**Affiliations:** ^1^ Department of Psychology, University of Innsbruck, Innsbruck, Austria; ^2^ Department of Psychology, University of Graz, Graz, Austria; ^3^ Department of Child and Adolescent Psychiatry, Psychotherapy and Psychosomatics, Medical University of Innsbruck, Innsbruck, Austria; ^4^ VAMED Clinic for Rehabilitation Montafon, Schruns, Austria

**Keywords:** childhood trauma, mental health, COVID-19, depression, psychological distress

## Abstract

**Background:**

Initial studies suggest that individuals with a history of traumatic life experiences, particularly childhood trauma, may be more susceptible to increased mental health problems in the wake of the COVID-19 pandemic.

**Methods:**

The present cross-sectional study compared the mental health status of three cohorts of university students before (2016), at the beginning (2020) and at the end (2022) of the COVID-19 pandemic. The students in each cohort were divided into two groups: those with self-reported moderate/severe childhood trauma (n = 126) and those with no/mild childhood trauma (n = 438).

**Results:**

Across all cohorts, students with moderate/severe childhood trauma consistently reported higher levels of psychological and physical stress compared to individuals with no/mild childhood trauma experiences. However, only the no/mild childhood trauma group exhibited an increase in mental health problems (i.e., heightened depressive symptoms and greater subjective impairment due to physical and psychological symptoms) during the COVID-19 pandemic. Furthermore, within the no/mild childhood trauma group, students in the 2022 cohort reported significantly higher psychological distress compared to those surveyed in 2020. In contrast, mental health scores among students with moderate/severe childhood trauma remained unchanged across the pre-pandemic and pandemic cohorts.

**Conclusions:**

The findings of this study do not support the hypothesis that the COVID-19 pandemic disproportionately exacerbated mental health problems in individuals with a history of moderate to severe childhood trauma. Instead, our results suggest that the pandemic’s impact on mental health was more pronounced in students with no or only mild childhood trauma.

## 1 Introduction

The COVID-19 pandemic has been a major challenge worldwide, with numerous studies documenting an increase in mental illness (e.g., depression, anxiety disorders and other stress-related illnesses) following the outbreak of the pandemic [see, for a systematic review (1) or (2)]. Students in particular represent a vulnerable group who are exposed to a variety of stressors such as social, financial and emotional challenges, in addition to academic demands (3, 4). Even before the COVID-19 pandemic, students reported high levels of stress (5). Persistent stress can lead to poorer academic performance, higher dropout rates (6), and is one of the most important risk factors for mental (and physical) health (3).

The wide range of stress factors makes students particularly susceptible to mental illness. For instance, an international survey of universities found that more than a third of their students already met the DSM diagnostic criteria for depression or anxiety disorders (3), highlighting the vulnerability of this population. The shift to digital distance learning during the COVID-19 pandemic, exacerbated these challenges, leading to a further significant increase in anxiety symptoms, depression and post-traumatic stress disorder (PTSD) among students (7, 8). According to a study by Karasmanaki and Tsantopoulos (9), the shutdown of universities and the associated changes in university life constituted a massive disruption for students and had a significant impact on their well-being.

In the public media and numerous articles, the COVID-19 pandemic is sometimes referred to as a traumatic experience or collective trauma [see, for example (10)]. However, there is currently no single valid definition of “trauma”. The eleventh revision of the International Classification of Diseases, ICD-11 (11), defines a traumatic event as an “extremely threatening or distressing event”, which can be a single event or a series of events. The Diagnostic and Statistical Manual of Mental Disorders, Fifth Edition (DSM-5) operationalizes traumatic events as direct or indirect confrontation with death, whether actual or threatened, as well as with serious injury or sexual violence (12). Although the COVID-19 pandemic cannot be classified as a traumatic event according to these criteria, many researchers argue that it fulfills the requirements for trauma classification and consider it a traumatic event [e.g. (13)]. A study on COVID-19 and trauma-related symptoms in Austrians in spring 2021 revealed a prevalence of trauma-related symptoms of 18.1%, which, according to the authors (14), is higher than expected based on international data. The study found that symptoms of re-experiencing and, to a lesser extent, avoidance were more common in COVID-19 cases, while dissociative symptoms, hyperarousal, and negative mood were less prevalent. Younger people in particular showed more pronounced trauma-related symptoms, which the authors attribute to less life experience and fewer stress management skills (14).

A French study (15) comparing anxiety and depression symptoms in students and non-students at three points during the pandemic found that students reported more depressive symptoms during the initial nationwide lockdown, comparable levels during the relaxation phase, and a subsequent increase during the second lockdown. In addition, anxiety symptoms were generally more prevalent in students during the pandemic than in people who were not studying. These findings suggest that the governmental restrictions disproportionately affected students compared to their peers who were not in academic education (15). A meta-analysis by Jia et al. (16) further confirmed that students are more susceptible to depression and anxiety than the general population and healthcare professionals. Many studies also identify female gender as another important risk factor for pandemic-related psychological reactions including anxiety disorders, depression, and post-traumatic stress symptoms and PTSD [e.g., (17)], although the meta-analysis by Peng et al. (18) points to a certain heterogeneity in these findings.

Regardless of the discussion about whether or not the COVID-19 pandemic is formally classified as a traumatic event, preliminary research suggests that individuals with previous traumatic life events, particularly childhood trauma, may be more vulnerable to increased anxiety, depression and post-traumatic stress symptoms in the wake of the COVID-19 pandemic (19–21). It is known that individual exposure to environmental stressors during early life impairs adaptive coping strategies and thus increases vulnerability to future stressors (22). Many studies therefore emphasize the importance of considering people’s past traumas when assessing mental health during the COVID-19 pandemic (20).

A significant proportion of the population has a history of trauma (23), and students in particular frequently report childhood trauma (24, 25). A distinction is made between different types of childhood trauma, including physical and emotional abuse, sexual abuse or neglect (26). Childhood trauma is considered a risk factor that can lead to a variety of mental disorders (27), and both to decreased mental as well as physical health (28). Research shows that individuals with traumatic childhood experiences are more susceptible to increased mental health problems in later stressful situations (29).

Studies investigating the impact of childhood trauma on mental health during the COVID-19 pandemic have shown that childhood trauma and adverse childhood experiences negatively affected mental health (30, 31). According to Stanislawski et al. (32), the experience of emotional abuse in childhood was associated with higher levels of depression and PTSD in students during the pandemic. Similarly, Xie et al. (33) found a positive association between childhood trauma, pandemic-related psychological distress, and depression, anxiety and stress. Conversely, secure attachment in childhood may serve as a protective factor against the development of psychological symptoms, according to Bussone, Pesca, Tambelli, and Carola (34).

Therefore, the present cross-sectional study has two primary aims: to compare the mental health status of university students before and during the two-year pandemic, and to investigate whether students with higher levels of childhood trauma were more vulnerable to COVID-19 stressors and reported more mental health problems during the COVID-19 pandemic.

## 2 Materials and methods

### 2.1 Study procedure and participants

The sample consists of three cohorts of German-speaking students from different faculties in Austria and Germany. The students were recruited via social networks, university courses and the mailing lists of the University of Innsbruck and Graz. The pre-COVID group was surveyed between September and December 2016 and a second and third survey of students was conducted during two distinct phases of the COVID-19 pandemic. The survey for the second cohort was started in May 2020, was stopped during the semester break and was completed in mid-November 2020. This period coincided with initial experiences of restrictive measures and university closures, necessitating a transition from face-to-face to distance learning. The third cohort was surveyed in June 2022. The third survey period was characterized by a substantial easing of restrictions. Nevertheless, many university courses continued to be held online for various reasons.

This study was approved by the ethics committees of the University of Graz and the University of Innsbruck. All participants provided informed consent prior to their study inclusion.

A total of n = 747 students participated in the online surveys. Only students between the ages of 18 and 26 were included in the analyses. Students who were already working full-time and were not registered as “actively studying” at the time of the survey were excluded from the study. Participants with incomplete questionnaires were also excluded. The final sample consisted of 564 students (pre-COVID group: n = 175; COVID-Year 2020 group: n = 200; COVID-Year 2022 group: n = 189). Using the Bernstein, Fink, Handelsman, and Foote (35) cut-off scores, students in each cohort were categorized into a group with moderate/severe childhood trauma (n = 126) and a group with no/mild childhood trauma (n = 438).

### 2.2 Measures

#### 2.2.1 Beck’s depression inventory

The BDI-II (36) was administered to all participants to assess the severity of depressive symptoms. Participants self-reported a variety of current depressive symptoms, rating each on a 4-point Likert scale from 0 (absent or mild) to 3 (severe). The total BDI-II score is calculated by summing all 21 item scores and thus ranges from 0 to 63. Higher scores indicate more severe depressive symptoms. A review of the psychometric properties of the BDI-II (37) reported internal consistency around 0.9 and retest reliability ranging from 0.73 to 0.96. The review also found a high overlap (0.66 to 0.86) between the construct measured by BDI-II and that of other widely used depression scales, such as the Center for Epidemiologic Studies of Depression Scale (CES-D) and, the Hamilton Depression Rating Scale (HAM-D). For the German version of the BDI-II Kühner et al. (38) reported Cronbach’s alphas of at least 0.84 across different samples, along with significant and strong correlations with similar measures (ranging from 0.72 to 0.89 across different samples). In the current study Cronbach’s alpha was also calculated to assess reliability. The internal consistency of the BDI-II in this sample is high, with α = 0.89.

#### 2.2.2 Modified impact of events scale – COVID-19

A modified version of the IES-R (39) was used to measure the traumatic impact of the COVID-19 pandemic. The IES-R, a 22 items scale, capture the subjective distress experienced in the past seven days as a result of a traumatic event. It contains three subscales: Avoidance (8 items), Hyperarousal (7 items), and Intrusion (7 items), which correspond to DSM-IV criteria B, C, and D for PTSD. Each item is scored on a 4-point scale ranging from ‘not at all’ (0), ‘rarely’ (1), ‘sometimes’ (3) to ‘often’ (5), with higher scores indicating a greater psychological impact of the traumatic situation. The original IES-R instructions were slightly adapted to specifically address the COVID-19 situation. Items referring to the past, were changed to the present tense, as the COVID-19 pandemic was still ongoing during data collection.

The internal consistency of the modified version of the IES-R, used in the current study to measure the traumatic impact of the COVID-19 pandemic was high, with Cronbach’s α = 0.90.

#### 2.2.3 Childhood trauma questionnaire

The CTQ [35; German version by Klinitzke, Romppel, Häuser, Brähler, and Glaesmer (40)] is a self-report measure designed to retrospectively assess the occurrence and severity of child maltreatment and abuse (childhood trauma). The 28 items cover five subscales related to childhood trauma, namely sexual abuse, emotional abuse, physical abuse, emotional neglect, and physical neglect. Three additional questions assess whether participants minimize or deny their childhood trauma. Items are rated on a 5-point Likert scale from 1 (not at all) to 5 (very often) and summed to a global score. The following cut-off scores for the presence of moderate/severe childhood trauma, taken from Bernstein et al. (35), were used: ≥13 for emotional abuse, ≥10 for physical abuse, ≥8 for sexual abuse, ≥15 for emotional neglect, and ≥10 for physical neglect. The German version of the CTQ showed high internal consistency for all scales (Cronbach’s α: 0.80 – 0.89), except for physical neglect, Cronbach’s α: 0.55), in a sample of 2500 psychiatric patients (40). The established factor structure (i.e., sexual, physical, and emotional abuse, as well as physical and emotional neglect) was replicated by means of confirmatory factor analysis. Only low to moderate correlations between the CTQ and self-report measures for anxiety and depression on the Patient Health Questionnaire-4 (PHQ-4P) could be found. In the present study, the internal consistency of the CTQ global score was high, with a Cronbach’s α = 0.91.

#### 2.2.4 Symptom checklist 90 revised

The SCL-90-R [ (41); German version (42); 90 items] is a self-report questionnaire assessing subjective impairment caused by physical and psychological symptoms within the past seven days. The frequency and intensity of the symptoms are rated on a 5-point Likert scale from 0 (“not at all”) to 4 (“very strongly”). The Global Severity Index (GSI) calculated as the mean of all items, serves as an overall measure of psychological distress. An evaluation of the psychometric properties of the German version of the SCL-90-R (43) showed high internal consistency for the Global Severity Index (Cronbach’s α = 0.97) and all original subscales (Cronbach’s α = between 0.80 -0.90). Significant correlations between the subscales of the SCL-90 and other scales, such as the General Health Questionnaire (GHQ-12), ranged from 0.45 to 0.72 and provide evidence of concurrent validity. However, the original nine-factor and subsequent two factor model could not be replicated. In the current study, Cronbach’s alpha for the GSI was high, with α = 0.97.

### 2.3 Statistical analysis

Descriptive data are presented as means (M) and standard deviations (SD). Pearson’s chi-square tests were used to compare the demographic characteristics (gender and childhood trauma) of the three cohorts (pre-COVID [first cohort], COVID-Year 2020 [second cohort], COVID-Year 2022 [third cohort]). A univariate analysis of variance (ANOVA) was used to examine the age differences between the three cohorts of students.

Furthermore, univariate two-factor analyses of covariance (ANCOVAs) were conducted for BDI-II, SCL-90-R, and CTQ scores, with cohort (pre-COVID, COVID-Year 2020, and COVID-Year 2022), and childhood trauma (no/mild childhood trauma vs. moderate/severe childhood trauma) as fixed factors, and age as a covariate. Similarly, an ANCOVA was used to analyze differences in the mIES-R scores between the two COVID-19 groups (COVID-Year 2020 and COVID-Year 2022), again with age as covariate. Consistent with the research questions of the present study, the relevant effects of interest in these analyses were the main effects of cohort (differences in the overall symptom level) and the two-way interaction between cohort and childhood trauma. *Post-hoc* pairwise comparisons with Bonferroni-corrections were conducted to explore significant interactions effects. Effect sizes in ANCOVAs are reported as partial eta-squared (ηp^2^). Moreover, for significant ANCOVA effects we additionally report adjusted means and 95% confidence intervals of the means (CIs). All analyses were performed using the SPSS software (version 26) with a significance level of α = .05 (two-tailed).

To determine the achieved power of the significant ANCOVA results, *post-hoc* power analyses were conducted using G*Power 3.1 (44). First, we converted the obtained partial eta-squared effect sizes to Cohen’s f effect sizes. For the power analyses, we specified an α-level of 0.05, a total sample size of 564, and the number of groups and covariates. The analyses demonstrated that the present study was adequately powered to detect small effects in the BDI-II, SCL-90-R GSI, and CTQ scores.

## 3 Results

### 3.1 Sample characteristics

The majority of the sample was female and reported no/mild childhood trauma. There were no significant differences in the distribution of gender (χ^2^(2, n = 564) =2.054, *p* = .358) or the number of students across the two childhood trauma groups (χ^2^(2, n = 564) = 4.090, *p* = .129) in the three cohorts. However, there was a significant difference in the age of students among the three cohorts (F(2,561) = 77.174, *p* <.001, ηp² = .215), with the oldest students belonging to the third cohort, followed by the second and the first cohort (all *p*’s <.001).

Demographic data are presented in **Table 1**.

**Table 1:** Demographic data of the three student cohorts.

	pre-COVID 2016 n = 175	COVID-Year 2020 n = 200	COVID-Year 2022 n = 189
Age [Years], mean (SD)	20.8 (2.0)	22.3 (1.9)	23.4 (1.9)
Sex			
Women, n (%)	141 (81%)	150 (75%)	142 (75%)
Men, n(%)	34 (19%)	50 (25%)	47 (25%)
Moderate/severe childhood trauma, n (%)	31 (18%)	43 (22%)	50 (26%)

**Table 2** summarizes the descriptive statistics for the four clinical scales at the three measurement periods, separately for the two childhood trauma groups.

**Table 2:** Means and standard deviations (in parentheses) for the four clinical scales.

	pre-COVID 2016		COVID-Year 2020		COVID-Year 2022	
	no/mild childhood trauma *(n* = 144)	moderate/severe childhood trauma *(n* = 31)	no/mild childhood trauma *(n* = 157)	moderate/severe childhood trauma *(n* = 43)	no/mild childhood trauma *(n* = 139)	moderate/severe childhood trauma *(n* = 50)
**BDI**	5.7 (5.5)	11.1 (7.2)	7.5 (5.6)	13.2 (11.5)	9.8 (7.9)	13.3 (8.8)
**SCL-GS**	0.4 (0.3)	0.7 (0.5)	0.4 (0.3)	0.8 (0.6)	0.59 (0.48)	0.86 (0.66)
**CTQ_GS**	45.3 (4.5)	58.9 (7.4)	45.7 (4.2)	68.3 (17.8)	46.4 (4.1)	65.7 (12.8)
**mIES-R**			35.6 (9.5)	38.5 (12.5)	38.7 (11.3)	40.6 (10.5)

The table shows means (standard deviations). BDI, Beck Depression Inventory, SCL 90 R-GS, Symptom-Checklist 90 Revised Global Score; CTQ_GS, Child Trauma Questionnaire - Global Score; mIES-R, Modified Impact of Event Scale – COVID-19.

### 3.2 The impact of COVID-19 pandemic and childhood trauma on depression scores

The ANCOVA using the BDI-II as the dependent variable revealed no significant interaction between the cohort and childhood trauma experiences (F(2,557) = .737, *p* = .479).

However, there was a significant main effect of childhood trauma (F(1,557) = 43.512, *p* <.001, ηp^2^ =.072), with individuals who experienced moderate/severe trauma exhibiting significantly higher BDI-II scores compared to those who experienced no/mild childhood trauma. Adjusted means were 7.66, 95% CI [6.99, 8.33], for the no/mild trauma group and 12.53, 95% CI [11.25, 13.82], for the moderate/severe trauma group.

Furthermore, a significant main effect of cohort was identified (F(2,557) = 8.097, *p* <.001, ηp^2^ = .028). After adjusting for age, Bonferroni-corrected *post-hoc* tests revealed that the pre-COVID group had the lowest BDI-II score (adjusted mean = 7.92, 95% CI [6.46, 9.37]), differing significantly from both the COVID-Year 2020 cohort (*p* = .033; adjusted mean = 10.38, 95% CI [9.17, 11.59]) and the COVID-Year 2022 cohort (*p* <.001; adjusted mean = 11.99, 95% CI [10.77, 13.21]). There was no significant difference in the BDI-II scores between the COVID-Year 2020 and COVID-Year 2022 cohorts (*p* = .201).

After adjusting for age, Bonferroni-corrected *post-hoc* tests revealed significant differences in the BDI-II scores between the three cohorts in the no/mild childhood trauma group. The pre-COVID group (adjusted mean = 5.28, 95% CI [4.04, 6.52]) had lower scores than both the COVID-19 Year 2020 cohort (*p* = .024; adjusted mean = 7.55, 95% CI [6.43, 8.67]) and the COVID-19 Year 2022 cohort (*p* <.001; adjusted mean = 10.14, 95% CI [8.90, 11.38]). Furthermore, the COVID-19 Year 2020 cohort had lower BDI-II scores than the COVID-19 Year 2022 cohort (*p* = .007).

No significant differences in the BDI-II scores were observed between the three cohorts in the moderate/severe childhood trauma group.

*Post-hoc* analyses revealed an achieved statistical power of 0.96 or higher for both significant main effects in the ANCOVA.

**Figure 1:**
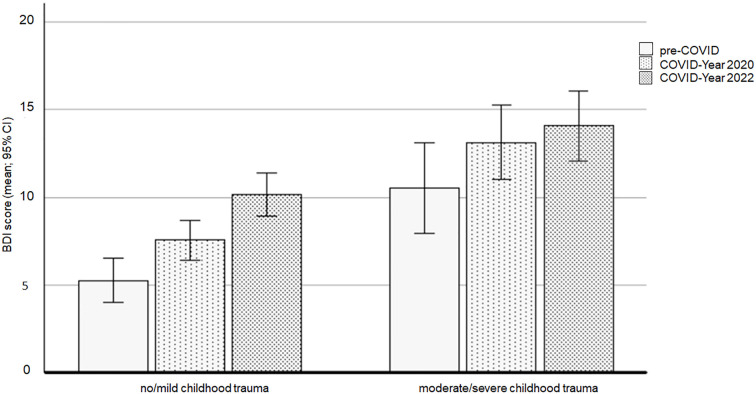
BDI scores affected by the COVID-19 pandemic and the experience of childhood trauma. BDI, Beck’s Depression Inventory.

**Figure 1** presents the means and 95% confidence intervals (CIs) of the BDI-II scores for the three cohorts and the two childhood trauma groups.

### 3.3 The impact of COVID-19 pandemic and childhood trauma on psychological distress

The ANCOVA using the SCL-90-R Global Severity Index (GSI) showed no significant interaction between cohort and childhoodtrauma (F(2,557) = .190, *p* = .827). However, there was a significant main effect of trauma (F(1,557) = 44.642, p <.001, ηp^2^ = .074), with significantly higher GSI score in the moderate/severe childhood trauma group compared to the no/mild childhood trauma group (*p* <.001). Adjusted means were 0.48, 95% CI [0.44, 0.52], for the no/mild trauma group and 0.78, 95% CI [0.70, 0.86], for the moderate/severe trauma group.

A significant main effect of cohort was also found (F(2,557) = 4.965, *p* = .007, ηp^2^ = .018). *Post-hoc* tests indicated that the GSI score was significantly higher in the COVID-Year 2022 cohort (adjusted mean = 0.74, 95% CI [0.66, 0.81]) compared to the pre-COVID group (*p* = .012; adjusted mean = 0.56, 95% CI [0.47, 0.65]) and the COVID-Year 2020 cohort (p = .038; adjusted mean = 0.60, 95% CI [0.53, 0.68]). There was no significant difference in GSI scores between the pre-COVID and the COVID-Year 2020 cohorts (*p* = 1.000).

Further analysis within each childhood trauma group revealed significant differences in GSI scores between the three cohorts in the no/mild childhood trauma group. Specifically, the COVID-Year 2022 cohort (adjusted mean = 0.60, 95% CI [0.52, 0.68]) had higher scores than both the pre-COVID cohort (*p* = .002; adjusted mean = 0.40, 95% CI [0.33, 0.48]) and the COVID-Year 2020 group (*p* = .004; adjusted mean = 0.43, 95% CI [0.36, 0.50]). No significant differences in GSI scores were observed between the cohorts in the moderate/severe childhood trauma group.

**Figure 2:**
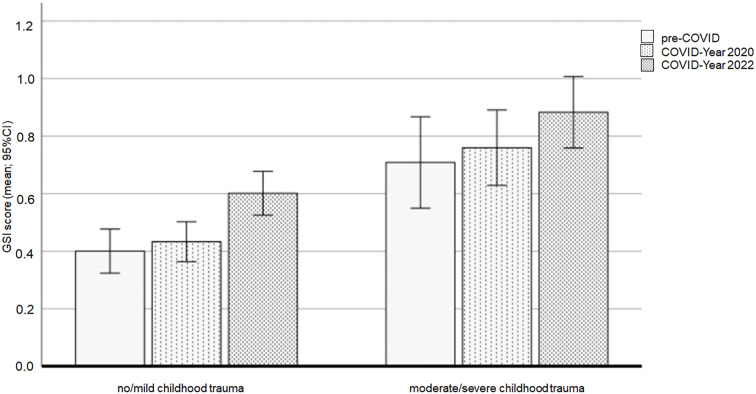
GSI scores affected by the COVID-19 pandemic and the experience of childhood trauma. Note: GSI = SCL-90 General Severity Index.

**Figure 2** presents the means and 95% confidence intervals (CIs) of the GSI scores for the three cohorts and the two childhood trauma groups.

*Post-hoc* analyses revealed an achieved statistical power of at least 0.85 for both significant main effects in the ANCOVA.

### 3.4 Differences between the cohorts in the childhood trauma scores

The ANCOVA using the CTQ global score showed a significant main effect of cohort (F(2,557) = 11.731, *p* <.001, ηp^2^ = .040), a main effect of childhood trauma (F(1,557) = 579.060, *p* <.001, ηp^2^ = .510), and a significant interaction between cohort and childhood trauma (F(2,557) = 10.561, *p* <.001, ηp^2^ = .037).

Overall, there was a significant difference in the CTQ scores between the three cohorts. The pre-COVID cohort had the lowest CTQ global score (adjusted mean = 52.22, 95% CI [50.70, 53.73]) differing significantly from both the COVID-Year 2020 cohort (*p* <.001; adjusted mean = 57.01, 95% CI [55.75, 58.27]) and the COVID-Year 2022 cohort (*p* = .001; adjusted mean = 55.97, 95% CI [54.70, 57.24]). There was no significant difference in CTQ scores between COVID-Year 2020 and COVID-Year 2022 cohorts (*p* = .759).

As expected, given that childhood trauma groups were derived from CTQ scores, there was a significant difference in CTQ scores between the childhood trauma groups, with lower scores in the no/mild childhood trauma group (adjusted mean = 45.82, 95% CI [45.12, 46.52]) compared to the moderate/severe childhood trauma group (*p* <.001; adjusted mean = 64.31, 95% CI [62.97, 65.65]).

To further understand the interaction between cohort and childhood trauma, separate Bonferroni-corrected *post-hoc* analyses were conducted for each trauma group. In the moderate/severe childhood trauma group, these tests revealed significant differences in CTQ global scores between the cohorts. The pre-COVID cohort (adjusted mean = 59.04, 95% CI [56.37, 61.71]) had lower scores compared to the COVID-Year 2020 cohort (*p* <.001; adjusted mean = 68.31, 95% CI [66.08, 70.53]) and the COVID-Year 2022 cohort (*p* <.001; adjusted mean = 65.59, 95% CI [63.47, 67.70]). No difference was observed between the COVID-Year 2020 and the COVID-Year 2022 cohorts (*p* = .249). In the no/mild childhood trauma group, there were no significant differences in CTQ global scores between the cohorts (all *p*’s >.05).

**Figure 3:**
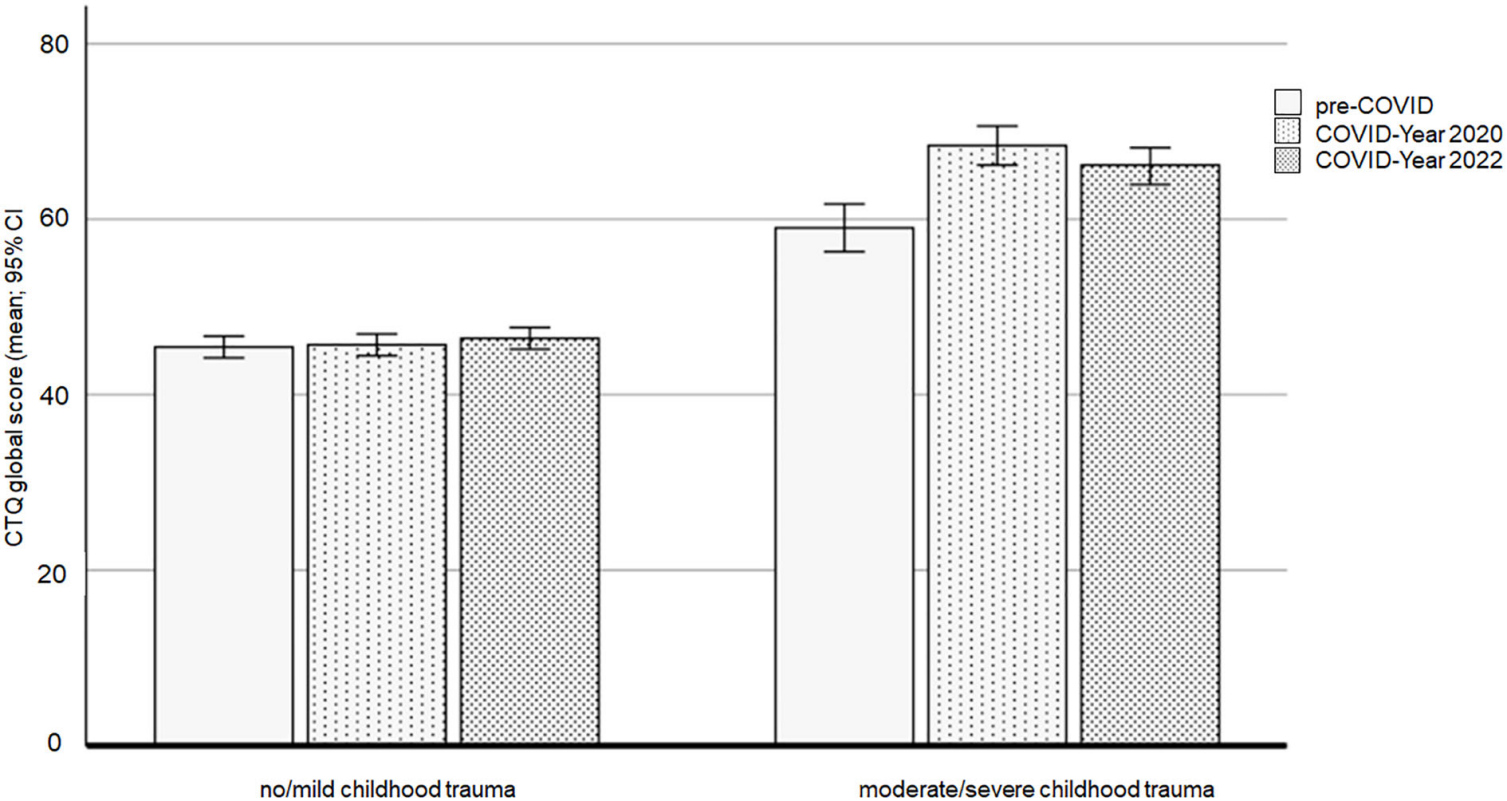
CTQ global scores affected by the COVID-19 pandemic and the experience of childhood trauma. Note: CTQ = Child Trauma Questionnaire.

**Figure 3** shows the means and 95% confidence intervals (CIs) of the CTQ global scores for the three cohorts and the two childhood trauma groups.

*Post-hoc* analyses demonstrated an achieved statistical power of at least 0.99 for both the significant main effects and the interaction effect of CTQ scores.

### 3.5 The traumatic impact of the COVID-19 pandemic

The modified IES-R (mIES-R) was used to assess the traumatic impact of the COVID-19 pandemic in the two COVID-19 cohorts. The results revealed a marginal main effect of childhood trauma (F(1,384) = 3.598, *p* = .059, ηp^2^ = .009), with a trend toward higher mIES-R scores in the moderate/severe childhood trauma cohort compared to the no/mild childhood trauma cohort.

There was also a marginal effect of cohort (F(1,384) = 3.654, *p* = .057, ηp^2^ = .009), with lower mIES-R scores in the COVID-Year 2020 cohort (adjusted mean = 37.11, 95% CI [35.28, 38.94]) compared to the COVID-Year 2022 cohort (adjusted mean = 39.62, 95% CI [37.86, 41.39]). The interaction between cohort and childhood trauma did not reach significance (F(1,384) = .143, *p* = .705). Additionally, Bonferroni-corrected *post hoc* tests showed that mIES-R scores increased significantly over time only in the no/mild childhood trauma cohort (*p* = .019; adjusted mean for COVID-Year 2020 = 35.66, 95% CI [33.97, 37.36] and adjusted mean for COVID-Year 2022 = 38.66, 95% CI [36.86, 40.46]).

**Figure 4:**
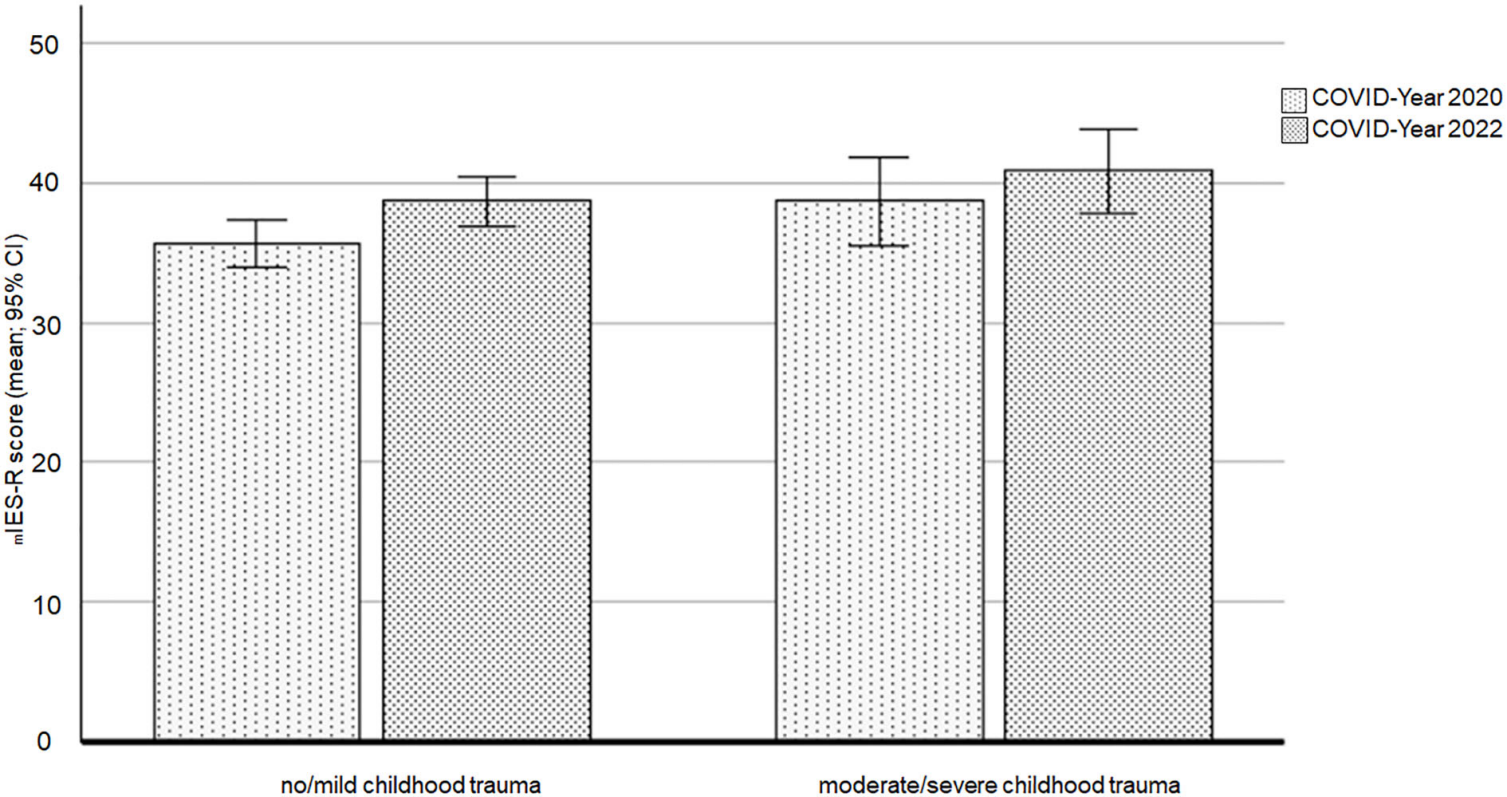
Impact of the COVID-19 pandemic on the two childhood trauma groups across the two COVID-19 cohorts. Note: mIES-R = Modified Impact of Event Scale – COVID-19.

**Figure 4** presents the means and 95% confidence intervals (CIs) for the mIES-R scores for the three cohorts and the two childhood trauma groups.

## 4 Discussion

In general, individuals with a history of childhood trauma have an increased risk developing mental disorders including post-traumatic stress symptoms, depression, anxiety disorders, and substance use disorders (30, 32, 33). Consistent with this, the current study found that in all three cohorts, students with moderate/severe childhood trauma reported higher levels of depression (BDI-II) and greater subjective impairment due to both physical and psychological symptoms (SCL-90-R (GSI) compared to those with no/mild childhood trauma.

Remarkably, differences in mental health between the three cohorts were observed only in the no/mild childhood trauma group. Students in the Pre-Covid cohort had significantly lower levels of depressive symptoms and subjective impairment due to physical and psychological symptoms than the two cohorts surveyed during the pandemic (2020 and 2022). This suggests, that the increase in mental health problems during the COVID-19 pandemic was primarily observed in students with no/mild childhood trauma, while those with pre-existing moderate to severe childhood trauma histories showed relatively consistent high psychological distress levels regardless of the pandemic context.

Even prior to the COVID-19 pandemic, young adults, particularly students, experienced elevated levels of psychological distress (5). The pandemic introduced additional stressors, burdens, and challenges (45). Several studies have reported increased mental health symptoms and illnesses among students during the pandemic [e.g., (46, 47)]. Longitudinal studies have also demonstrated an increase in perceived psychological distress over the course of the pandemic (18, 48), or at least persistently high levels even after restriction eased (49). In this context, Goral, Lahad, and Aharonson-Daniel (50) proposed that psychological stress becomes more pronounced and severe when a traumatic experience extends over a longer period of time. Continuous exposure to stressors makes it more difficult to cope with stress, and both vulnerability and sensitivity to stress increase (50, 51). The findings of this study are consistent with those of previous research, which showed that students in the COVID-Year 2022 cohort reported more psychological distress due to the COVID-19 pandemic (mIES-R scale) than those in the COVID-Year 2020 cohort.

In contrast, no significant differences in depression (BDI-II) or SCL-90-R scores were found between the pre-Covid cohort and the two pandemic cohorts among students with moderate/severe childhood trauma. Furthermore, these students showed no change in the mIES-R scores between 2020 and 2022. These results are consistent with a study from Russo et al. (52), who found that in individuals with adverse childhood experiences (ACEs), pandemic-related stressors primarily impacted those with less emotion regulation deficits. In individuals with ACEs and higher emotion regulation deficits, the added stress caused of the COVID-19 pandemic did not result in further mental health deterioration, potentially due to a ceiling effect. Moreover, individuals with severe trauma experiences often perceive relationships as a source of threat (53). Consequently, social distancing and isolation during lockdowns may have had less impact on them. Some research on attentional bias supports this notion, suggesting that individuals with prior trauma exposure may be more avoidant of threat cues or have a higher threshold for perceiving them, leading to less fear or distress related to the pandemic [e.g., (54, 55)]. In contrast, other studies have found that past trauma exposure is associated with an attentional bias towards threat, potentially contributing to increased mental health problems during the COVID-19 pandemic (56, 57).

### 4.1 Limitations

A limitation of the current study is the cross-sectional cohort design, which prevents a direct examination of the trajectory of mental health problems in individuals with a history of childhood trauma. However, so far, longitudinal studies on this topic have yielded inconsistent results. Some studies suggest that individuals with pre-existing mental health diagnosis and/or a history of childhood trauma may experience worsening symptoms during the pandemic [e.g., (58, 59)], while others, particularly those involving adults receiving treatment for PTSD, show less fear, distress, and trauma reminders [e.g., (60, 61)]. This latter effect is particularly pronounced when controlling for complex PTSD symptoms (21).

In addition, the three cohorts in this study were not perfectly matched and differed in age. Moreover, the pre-COVID cohort had lower global CTQ scores than the two COVID cohorts among students with moderate/severe childhood trauma. Since participants retrospectively assessed the severity of child maltreatment and abuse, recall bias is a potential concern, potentially leading to an overestimation of the relationship between adverse childhood experiences and poorer health outcomes (62).

Furthermore, the present study focused exclusively on university students, limiting the generalizability of the findings to other populations. However, the homogenous education level within the student sample is a strength, as it eliminates potential confounding effects of education. It is also important to note that the sample has a higher proportion of female students, which may have influenced the results. Finally, the online recruitment strategy may have introduced selection bias.

### 4.2 Practical and clinical implications of the study

The results of this study have significant practical and clinical implications for mental health professionals, university administrators, and policy makers. Young adulthood is a high-risk period for developing mental health problems (45, 46). It is crucial to offer support, such as stress management training, to students, especially during stressful periods like the COVID-19 pandemic. Effective stress management skills that promote adaptive emotion regulation and coping strategies, and enhance a sense of control, can protect against stress-related illness, unhealthy behaviors, and the long-term physical and psychological effects of stress (63). In a recent study we could demonstrate that a 7-week app-based passive psychoeducation program for stress management significantly improved adaptive emotion regulation strategies and coping styles, even in students with low clinically relevant psychopathological symptoms (64). Since passive psychoeducation does not use elements of active psychotherapies or require homework, it is an easily accessible and cost-effective self-guided intervention.

In the current study, students with moderate to severe childhood trauma consistently reported high levels of depression and psychological distress, regardless of the pandemic context. This underscores the need for specialized mental health services tailored to trauma-affected individuals. Trauma-informed care approaches, including cognitive-behavioral therapy (CBT), dialectical behavior therapy (DBT), and emotion regulation training, should be integrated into university counseling services to better support these students.

## 5 Conclusions

This study highlights the vulnerability of students to mental health problems during the COVID-19 pandemic. It specifically examined whether students with a history of childhood trauma were more susceptible to pandemic-related stressors and mental health issues. Comparing three student cohorts before and during the pandemic, the study confirmed previous findings that individuals with moderate or severe childhood trauma reported higher levels of psychological and physical distress than those with minimal or no trauma. However, the study did not find evidence that the pandemic disproportionately impacted the mental health of students with childhood trauma compared to those without. It is possible that individuals with pre-existing trauma exposure may engage in more avoidance of potentially threatening COVID-19-related information. Further research is needed to explore the complex interplay between the COVID-19 pandemic and its long-term effects on the mental health of individuals with a history of trauma.

## Data Availability

The datasets generated and/or analysed during the current study are available from the corresponding author upon reasonable request.
